# Ras and Gpa2 Mediate One Branch of a Redundant Glucose Signaling Pathway in Yeast

**DOI:** 10.1371/journal.pbio.0020128

**Published:** 2004-05-11

**Authors:** Ying Wang, Michael Pierce, Lisa Schneper, C. Gökçe Güldal, Xiuying Zhang, Saeed Tavazoie, James R Broach

**Affiliations:** **1**Department of Molecular Biology, Princeton UniversityPrinceton, New JerseyUnited States of America

## Abstract

Addition of glucose to starved yeast cells elicits a dramatic restructuring of the transcriptional and metabolic state of the cell. While many components of the signaling network responsible for this response have been identified, a comprehensive view of this network is lacking. We have used global analysis of gene expression to assess the roles of the small GTP-binding proteins, Ras2 and Gpa2, in mediating the transcriptional response to glucose. We find that 90% of the transcriptional changes in the cell attendant on glucose addition are recapitulated by activation of Ras2 or Gpa2. In addition, we find that protein kinase A (PKA) mediates all of the Ras2 and Gpa2 transcriptional effects. However, we also find that most of the transcriptional effects of glucose addition to wild-type cells are retained in strains containing a PKA unresponsive to changes in cAMP levels. Thus, most glucose-responsive genes are regulated redundantly by a Ras/PKA-dependent pathway and by one or more PKA-independent pathways. Computational analysis extracted RRPE/PAC as the major response element for Ras and glucose regulation and revealed additional response elements mediating glucose and Ras regulation. These studies provide a paradigm for extracting the topology of signal transduction pathways from expression data.

## Abstract 2

Inducing expression of most glucose responsive genes in yeast can be mimicked by activation of the small GTP-binding proteins Ras2 or Gpa2 even in the absence of a physiologic signal

## Introduction

Complex intracellular networks inform a cell's developmental and growth decisions in response to external nutrients or signaling molecules. Defining the topology of such networks has generally relied on combinations of genetic epistasis and biochemical techniques to establish the linear order of components that convey information on the presence of a particular stimulus. Generally, only one or a few endpoints, such as enhanced transcription of a responsive gene, are monitored in gauging the output of a pathway. More recently, global transcriptional analysis has allowed reseachers to capture the entire transcriptional output of a signaling process and assess the consequence of eliminating individual components of the signaling network on the entire response (Fambrough et al. 1999; Roberts et al. 2000). This approach has the potential to extract a complete description of a network from a relatively limited set of experimental perturbations.

We have used global transcriptional analysis to dissect the signaling network activated by glucose addition to yeast cells, with an emphasis on the role of the small GTP-binding proteins, Ras2 and Gpa2, in that signaling process. Addition of glucose to yeast cells growing on a nonfermentable carbon source induces a dramatic restructuring of the metabolic and transcriptional state of the cell (Johnston and Carlson 1992). At the metabolic level, the cell becomes reprogrammed for fermentative rather than oxidative growth. This involves the inactivation and repression of gluconeogenic enzymes and mitochondrially based oxidative phosphorylation processes and the induction of glycolytic enzymes. In addition, since yeast cells extract energy more efficiently from fermentable carbon sources, they are able to grow more rapidly and thus require an increase in the capacity for mass accumulation. This translates primarily into a need for increased protein synthetic capacity with an attendant increased production of ribosome components and other elements of the translational apparatus.

The dramatic change in the metabolic activity and protein synthesis capacity attendant on glucose addition to starved cells is accompanied, and driven in part, by a reprogramming of the transcriptional state of the cell (Johnston and Carlson 1992; DeRisi et al. 1997; Johnston 1999). Cells respond to glucose addition by repressing genes involved in the use of alternative carbon sources and in oxidative phosphorylation and by upregulating glucose-specific transport systems and glycolytic enzymes. Substantial work on glucose regulation of genes required for metabolism of alternate carbon sources, sometimes referred to as carbon catabolite repression, has identified a number of components of the network responsible for this repression and defined their interconnections (Gancedo 1998). For instance, an AMP-stimulated kinase, Snf1/Snf4, inactivates a repressor, Mig1, thereby allowing transcription of genes normally repressed in the presence of readily fermentable carbon sources, and upregulates Cat8, an activator of gluconeogenic genes (Carlson 1999). In addition, a number of transcriptional activators, such as Hap2/3/4, Adr1, etc., required for transcription of glucose-repressible genes, are inactivated by growth on fermentable carbon sources. Transcriptional upregulation of hexose transporters occurs by a glucose-induced degradation of Rgt1, a repressor of a number of glucose-induced genes (Johnston 1999). The mechanism by which glucose regulates genes needed for increased translational capacity is less clear, although Rap1 and, more recently, Sfp1 and Fhl1 have been implicated as activators responsible for increased expression of growth-related genes in response to glucose (Warner 1999; Jorgensen et al. 2002; Lee et al. 2002; Fingerman et al. 2003). However, it is not well defined whether the signal for such upregulation is the increased energy output or the presence of glucose per se.

The small GTP-binding proteins, Ras1 and Ras2, play a role in the cell's adaptation to glucose by coupling cyclic AMP (cAMP) production to the presence of glucose in the medium (Broach and Deschenes 1990; Tatchell 1993; Thevelein 1994). As in other organisms, yeast Ras proteins can transmit a regulatory signal by shuttling between an inactive GDP-bound form and an active GTP-bound form. In yeast, the GTP-bound Ras proteins stimulate adenylyl cyclase, encoded by *CYR1*, to yield an increase in intracellular cAMP levels (Toda et al. 1985). Addition of glucose to starved cells or cells growing on a nonfermentable carbon source yields within minutes a significant increase in intracellular cAMP concentrations, which rapidly decline to a level somewhat higher than that in prestimulated cells. This cAMP response to glucose is dependent on Ras. cAMP functions in yeast to liberate the yeast cAMP-dependent protein kinase A (PKA) catalytic subunit, encoded redundantly by *TPK1, TPK2,* and *TPK3,* from inhibition by the regulatory subunit encoded by *BCY1* (Toda et al. 1987). Active PKA can phosphorylate a number of proteins involved in transcription, energy metabolism, cell cycle progression, and accumulation of glycogen and trehalose (Broach and Deschenes 1990; Tatchell 1993; Thevelein 1994; Boy-Marcotte et al. 1998; Smith et al. 1998; Stanhill et al. 1999). Several epistasis experiments have suggested that in some cases Ras may also function upstream of a MAP kinase cascade in yeast, primarily to direct pseudohyphal growth under conditions of nutrient limitation (Mosch et al. 1996, 1999).

*GPA2,* a member of the Gα family of heterotrimeric G proteins, also regulates cAMP levels through a pathway parallel to Ras (Nakafuku et al. 1988). Gpa2 associates with a protein, encoded by *GPR1,* which is structurally related to seven-transmembrane, G-protein–coupled receptors and whose ligand may be fermentable sugars (Yun et al. 1997; Xue et al. 1998; Lorenz et al. 2000). Several lines of evidence suggest that Gpa2 activates adenylyl cyclase in a Ras-independent fashion. Overexpression of Gpa2 yields increased cAMP levels in the cell and an activated allele of Gpa2, even in a *ras2* background, induces phenotypes associated with activated PKA, such as heat-shock sensitivity, repression of Msn2/4-dependent transcription, induction of pseudohyphal development, and loss of cellular stores of glycogen and trehalose (Nakafuku et al. 1988; Lorenz and Heitman 1997). Reciprocally, *gpa2* is synthetically lethal with *ras2*, a phenotype that is reversed by inactivation of *PDE2,* the major cAMP phosphodiesterase in the cell (Kubler et al. 1997; Xue et al. 1998). Whether Gpa2 functions solely to modulate PKA or has other signaling functions has not been resolved.

To address the role of Ras and Gpa2 in reconfiguring the yeast cell's transcriptional framework in response to glucose and to define the signaling network associated with glucose signaling, we examined the global transcriptional response of cells to glucose and compared the response to that of cells following induction of activated alleles of these two G proteins. The results of this analysis indicate that the vast majority of the transcriptional remodeling the cell undergoes in response to glucose addition can be recapitulated by induction of Ras2 or Gpa2. However, much of this change can also be accomplished in the absence of signaling through cAMP. This indicates that glucose signaling of transcriptional reorganization proceeds through redundant, overlapping pathways, only one of which is regulated by Ras2 or Gpa2.

## Results

### Activation of Ras2 or Gpa2 Recapitulates Most Glucose-Induced Transcriptional Changes

In order to examine the role of Ras2 and Gpa2 in effecting transcriptional changes in the cell in response to glucose, we measured the global transcriptional response of yeast cells immediately following induction of an activated allele of *RAS2* or *GPA2* (designated RAS2* and GPA2* in the figures) and compared that to the changes following glucose addition to glycerol-grown cells. To focus on signaling events, rather than the transcriptional consequences of metabolic changes in the cell, we examined the transcriptional response as it changed immediately following addition of glucose. Similarly, to examine the effects of Ras2 or Gpa2 activation, we constructed *gal1* strains that carried an activated form of *RAS2* or *GPA2* under control of the galactose-inducible *GAL10* promoter. Since *gal1* strains cannot metabolize galactose, addition of galactose resulted in induction of the activated *RAS2* or *GPA2* allele and a small number of other galactose-inducible genes, but resulted in no changes in the metabolic state of the cell.

In a parallel set of experiments, we examined the transcriptional changes in response to glucose addition of yeast cells containing a PKA that is unresponsive to intracellular cAMP levels. The mutant PKA, referred to as *tpk-w*, lacks the regulatory subunit and two of the redundant catalytic subunits, with the third catalytic subunit crippled in its activity (Cameron et al. 1988). As a consequence, such strains possess constitutive, low-level PKA activity that is unresponsive to changes in cAMP levels in the cell. Thus, changes in cellular behavior dependent on modulation of PKA activity should be abrogated in this strain. The results of both sets of experiments are available in Table S1.

**Figure 1:**
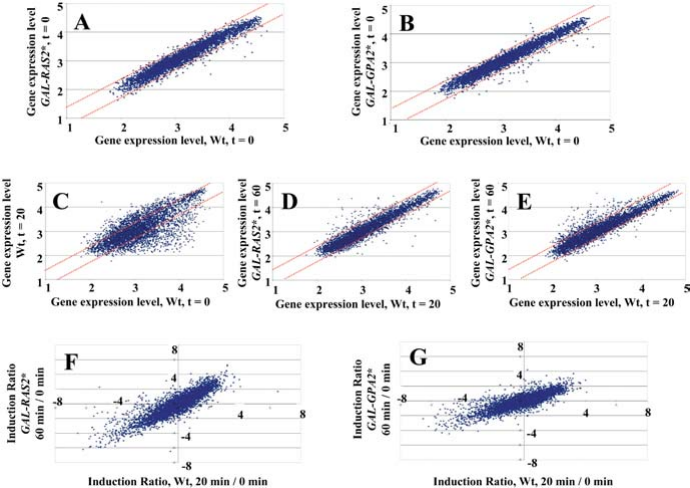
Glucose Stimulation and Ras2 or Gpa2 Activation Yield Similar Transcriptional Responses (A–E) Expression levels (represented as absolute intensity values from Affymetrix hybridization scans) of individual yeast genes (points) plotted for two different strains and conditions. Dotted red lines indicate 2-fold difference boundary. (A) Strain Y2864 (Wt) prior to glucose addition versus Y2866 (GAL-RAS2*) prior to galactose addition. (B) Strain Y2864 prior to glucose addition versus Y2876 (GAL-GPA2*) prior to galactose addition. (C) Strain Y2864 20 min after glucose addition versus 0 min after addition. (D) Strain Y2866 60 min after galactose addition versus Y2864 20 min after glucose addition. (E) Strain Y2876 60 min after galactose addition versus Y2864 20 min after glucose addition. Values are in log_10_. (F and G) Induction ratios (mRNA level at 60 min/mRNA level at 0 min) of genes in Y2866 (F) and Y2876 (G) versus induction ratios (mRNA level at 20 min/mRNA level at 0 min) for the same genes in Y2864. Values are in log_2_.

The results of comparing Ras2 activation to glucose addition, provided in Figure 1, indicate that most of the transcriptional changes in the cell immediately following addition of glucose to glycerol-grown cells are recapitulated by activation of Ras2. Prior to initiation of the experiment (during growth on glycerol), the expression pattern of all genes in the wild-type strain (W303 *gal1*) closely resembled that of the strain carrying the inducible activated Ras allele (W303 *gal1 GAL10*p*-RAS2* ^G19V^), with only 0.4% of the genes exhibiting greater than 3-fold differences in absolute expression levels (Figure 1A; *r* = 0.96). This reflects the isogenicity of the strains and indicates that the inducible *RAS2* ^G19V^ allele is not expressed under these conditions.

Addition of glucose to wild-type cells yields a substantial and rapid change in the transcriptional profile of the cell. By 20 min postaddition, 22% of all genes changed expression by greater than 3-fold and 41% changed expression by 2-fold, with essentially the same number of genes increasing as decreasing (Figure 1C). This dramatic change in the transcriptional profile was substantially recapitulated by activation of Ras. By 60 min postinduction, the profile of gene expression in the activated strain closely resembled that of the wild-type strain stimulated with glucose (Figure 1D; *r* = 0.94). Of those genes exhibiting a change in expression levels of at least 3-fold following addition of glucose, greater than 92% of those showed at least a 2-fold change in the same direction following activation of Ras2 (Figure 1F). Thus, since glucose yields activation of Ras2 and since Ras2 activation yields changes in transcription that are substantially similar to those observed following addition of glucose, we conclude that a major portion of the glucose signaling pathway regulating transcription can proceed through cAMP via Ras2.

Similar results emerge from analysis of expression changes following activation of Gpa2. Only 0.8% of all genes showed a greater than 3-fold difference in absolute expression levels between the wild-type strain and the strain carrying the inducible activated allele of Gpa2 during growth on glycerol (Figure 1B; *r* = 0.97). The pattern of expression at 1 h following activation of Gpa2 strongly resembles that at 20 min following addition of glucose to wild-type cells (Figure 1F; *r* = 0.93). However, the response following activation of Gpa2 under these conditions is not as robust as that following activation of Ras2 or addition of glucose. While the overall magnitude of the Ras2-induced response is essentially equivalent to that obtained by glucose addition, the overall magnitude of the Gpa2-induced response is only half that of the glucose-induced changes (Figure 1F and 1G). Nonetheless, although somewhat muted, the pattern of transcriptional change induced by Gpa2 closely resembles that induced by glucose. These results are consistent with the hypothesis that the major role of Gpa2 in the cell is modulation of cAMP in response to the presence of a fermentable carbon source.

### Redundant Signaling Pathways Control Glucose- Regulated Genes

**Figure 2:**
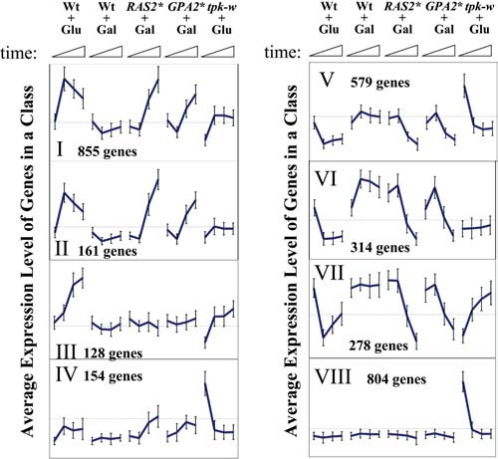
Expression Patterns of Clustered Genes Diagrams show the patterns of expression of genes in the classes (Roman numerals) listed in Table 1, which were clustered as described in Materials and Methods. Each line represents the average expression level of all genes in that cluster during the time course (20-min intervals over 1 h) in the strain and condition indicated. Absolute intensity values were normalized for each gene over all 32 conditions examined by subtracting the average expression level for that gene over the all conditions and dividing by the standard deviation for that gene. Thus, expression values (*y*-axis units) are represented as the standard deviations of each time point from the average expression value for each gene over the entire set of experiments. Error bars indicate the standard deviation in expression values of all genes in the cluster at the indicated timepoint. Abbreviations: Wt + Glu, glucose addition to strain Y2864; Wt + Gal, galactose addition to strain Y2864; RAS2* + Gal, galactose addition to strain Y2866; GPA2* + Gal, galactose addition to strain Y2876; *tpk-w* + Glu, glucose addition to strain Y2872.

To analyze the pattern of transcriptional response to glucose addition and cAMP induction, we used a partitional clustering algorithm to group genes on the basis of their behavior over all 32 samples analyzed (Heyer et al. 1999). Prior to clustering, the expression levels of each gene over the 32 samples were normalized by subtracting from each value the average expression of that gene over all experiments and dividing by the standard deviation of the expression values. This procedure emphasizes the pattern of response of each gene over the experiments, rather than the absolute levels of response. This process yielded 144 clusters ranging in size from seven to 506 members each. By hierarchical clustering (Eisen et al. 1998), these clusters were further organized into groups on the basis of the similarity of their patterns, yielding eight major classes exhibiting significant change in some respect over the course of the experiments. These classes, encompassing approximately 50% of all genes, are summarized in Table 1, and the corresponding pattern of expression is shown in Figure 2. The list of genes in each class is provided in Table S3.

**Table 1:**
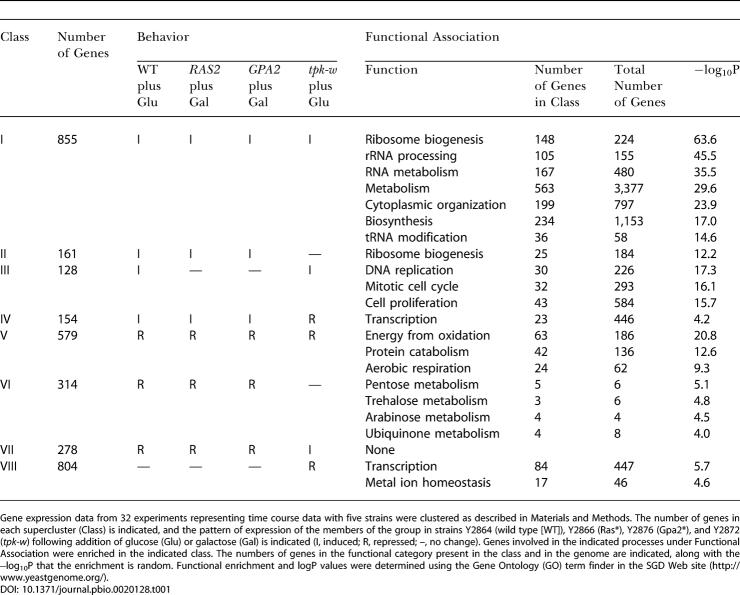
Functional Enrichment among Genes Clustered by Response to Glucose and Ras Activation

Gene expression data from 32 experiments representing time course data with five strains were clustered as described in Materials and Methods. The number of genes in each supercluster (Class) is indicated, and the pattern of expression of the members of the group in strains Y2864 (wild type [WT]), Y2866 (Ras*), Y2876 (Gpa2*), and Y2872 (*tpk-w*) following addition of glucose (Glu) or galactose (Gal) is indicated (I, induced; R, repressed; –, no change). Genes involved in the indicated processes under Functional Association were enriched in the indicated class. The numbers of genes in the functional category present in the class and in the genome are indicated, along with the −log_10_P that the enrichment is random. Functional enrichment and logP values were determined using the Gene Ontology (GO) term finder in the SGD Web site (http://www.yeastgenome.org/)

In general, glucose addition yielded a rapid change in expression of genes, which remained unchanged or tended back to starting conditions at later times. We interpret this behavior to indicate that the initial response, seen at the 20 min timepoint, generally represents the response of genes to the signal initiated by addition of glucose. The later deviation from that initial response represents either adaptation of the signaling process or readjustment of expression as a consequence of the change in metabolism of the cell. In contrast, gene expression in response to activation of Ras2 or Gpa2 generally showed a lag of 20 min, followed by a monotonic change in expression over the remainder of the experiment. This is consistent with the expectation that the effects of induction of Ras2 or Gpa2 can be seen only after the new activated protein is transcribed and translated. Further, since under these conditions no significant changes in metabolism occur, the change in expression is due solely to activation of the signaling pathway. This reinforces the notion that the initial response of the cell to glucose is a signaling response, since the pattern of this monotonic change at later times, following activation of Ras2 or Gpa2, generally matches the initial response of those genes to glucose addition.

If those genes induced by glucose and by activation of Ras2 are regulated by glucose solely through the Ras2–Gpa2–cAMP pathway, then we would anticipate that glucose-induced transcriptional alteration would be abrogated in a *tpk-w* strain. This is the case for a subset of glucose-affected genes (classes II and VI), indicating the existence of a glucose signaling pathway that relies solely on the Ras signaling pathway. Inversely, a subset of genes is activated (or repressed) by glucose in both the wild-type and *tpk-w* strains but is unaffected by activation of Ras2 or Gpa2, indicating the existence of a Ras2-independent glucose signaling pathway (class III). However, the vast majority of genes that respond to glucose are affected by Ras2 activation and also respond in the *tpk-w* background (classes I and V). This suggests that the majority of glucose-responsive genes are regulated by redundant pathways, one of which requires Ras2 and the other one(s) of which is Ras2 independent. Thus, the major transcriptional response of glucose addition diverges prior to activation of Ras2, but converges before gene activation. This is elaborated further in the Discussion.

### Ras and Gpa2 Signal Exclusively through PKA

**Figure 3:**
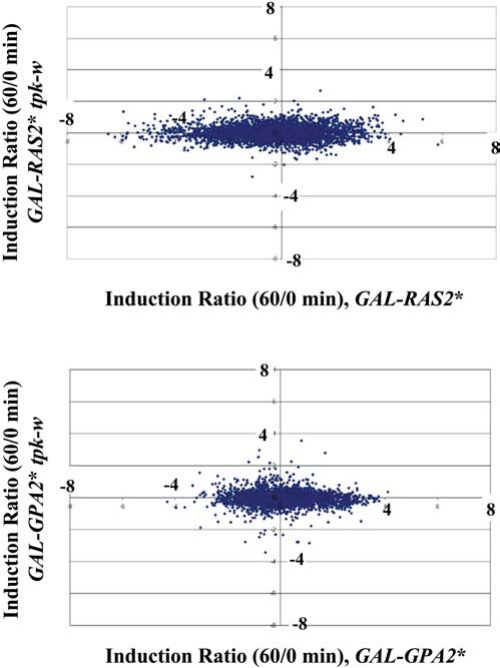
Ras and Gpa2 Affect Transcription Exclusively through PKA (Top) Induction ratios (mRNA level at 60 min/mRNA level at 0 min) of genes in strain Y2873 (*y*-axis) versus induction ratios (mRNA level at 60 min/mRNA level at 0 min) for the same genes in strain Y2866. Values are in log_2_. (Bottom) Similar analysis for strain Y2897 (*y*-axis) versus strain Y2876.

To assess the extent to which the effects on transcription of Ras2 activation are mediated by PKA, we examined the pattern of expression following activation of Ras2 in *tpk-w* cells compared to that in Tpk^+^ cells. For those genes whose induction or repression by Ras2 is exerted through PKA, the *tpk-w* mutations would be expected to abrogate that response. In Figure 3 we plot the change in expression of each gene 60 min after galactose addition to the *GAL10*p*-RAS2* ^V19^ *tpk-w* strain versus the change in expression of each gene 60 min after galactose addition to the *GAL10*p*-RAS2* ^V19^ strain. As evident, almost all genes fail to respond to Ras2 activation in the *tpk-w* background. Of the 789 genes (out of 4,037 analyzed) in this experiment whose expression increased by more than 2-fold at 60 min following addition of galactose to the *GAL10*p*-RAS2* ^V19^ strain, only 16 (2%) also showed increased expression through activation of Ras2 in the *tpk-w* background. Similarly, of the 1,121 genes whose expression decreased by more than 2-fold following activation of Ras2 in a wild-type background, only five (0.5%) also showed decreased expression in the *tpk-w* background. Repetition of these experiments using cDNA microarrays and direct Northern blot analysis of candidate genes failed to confirm that expression of any gene was altered by Ras induction in a *tpk-w* background (data not shown). Thus, we conclude that the entirety of the transcriptional response to Ras2 activation is mediated through PKA.

The results are similar for Gpa2 activation. As noted above, the response to Gpa2 activation is not as robust as that to Ras2 activation, and, as noted in Figure 3, the attenuation of the response to Gpa2 induction in a *tpk-w* strain is not as obvious as that seen with Ras2. Of the 444 genes in this experiment whose expression increased 2-fold or more in response to Gpa2 activation in a wild-type background, 75 (17%) also showed increased expression in the *tpk-w* background. Similarly, of the 831 genes whose expression decreased by 2-fold or more, 24 (3%) also showed decreased expression in the *tpk-w* background. However, multiple replicates of this experiment using cDNA microarrays failed to identify any gene consistently altered in transcription by Gpa2 in a *tpk-w* background. Thus, as with Ras, the vast majority, if not all, of Gpa2-responsive genes are regulated exclusively through PKA.

### Gpr1 Is Required for Efficient Glucose Response

**Figure 4:**
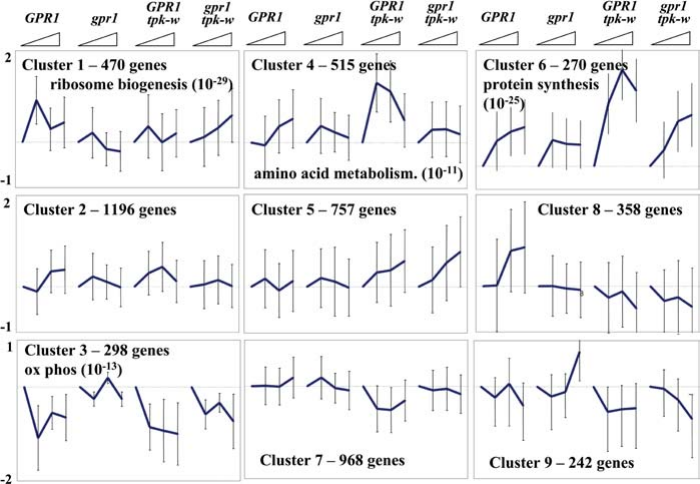
Loss of Gpr1 Diminishes the Glucose Response Diagrams show the patterns of expression of genes in clusters based on time course changes (20-min intervals over 1 h) in gene expression following glucose addition to the indicated strains (*GPR1*, Y2092; *gpr1*, Y3159; *GPR1 tpk-w*, Y2857; *gpr1 tpk-w*, Y3077). For clustering, absolute intensity values were normalized for each gene over all 12 conditions examined by subtracting the average expression level for that gene over all conditions and dividing by the standard deviation for that gene, but the plotted expression values (*y*-axis units) represent the average of the absolute intensity of expression (converted to log_2_) of all the genes in the cluster at the indicated timepoint. Error bars indicate the standard deviation in expression values of all genes in the cluster at the indicated timepoint. The number of genes in each cluster and any highly enriched function group (including the *p* value) are indicated in each graph.

*GPR1* encodes a protein structurally related to seven-transmembrane, G-protein–coupled receptors, and both biochemical and genetic evidence suggests it regulates Gpa2 activity in response to glucose (Xue et al. 1998; Kraakman et al. 1999; Lorenz et al. 2000). Accordingly, to assess the role of Gpr1 in the cell's transcriptional response to glucose, we examined the global transcriptional pattern of isogenic *GPR1* and *gpr1* strains at 20-min intervals following glucose addition to glycerol-grown cells. Further, to assess the extent to which Gpr1-mediated signaling was processed through PKA, we performed a similar time course experiment with isogenic *GPR1 tpk-w* and *gpr1 tpk-w* strains. The full set of data is available in Table S2. In both experiments we found that the overall transcriptional response (both induction and repression) was attenuated, although not eliminated, in the *gpr1* strain relative to the *GPR1* strain. For instance, for those genes whose expression changed by more than 50% following glucose addition to the *GPR1 TPK* strain, the average induction or repression ratio in the *gpr1* strain was approximately half that in the *GPR1* strain. K-means clustering of normalized data confirmed this general view (Figure 4). For instance, cluster 1, which included 470 genes highly enriched in those involved in ribosome biosynthesis, exhibited on average induced expression in the *GPR1 TPK* strain following glucose addition, but no induction in the *gpr1 TPK1* strain. Similar results were observed for genes in cluster 8, and induction of genes in clusters 4 and 6 was attenuated in the *gpr1* strain compared to that in the *GPR1* strain. Thus, these results are consistent with the hypothesis that Gpr1 participates in glucose signaling, but is not the sole mediator of that signaling.

The time course data from the *tpk-w* strain suggest that Gpr1 might affect multiple glucose signaling pathways. If a Gpr1-initiated signal were transmitted solely through PKA, then the pattern of gene expression following glucose addition to the *gpr1 tpk-w* strain would be essentially identical to that observed in the *GPR1 tpk-w* strain. While the correlation between the expression patterns of *gpr1 tpk-w* and *GPR1 tpk-w* (*r* = 0.73) is higher than that between *gpr1 TPK* and *GPR1 TPK* (*r* = 0.65), the patterns of expression of *gpr1 tpk-w* and *GPR1 tpk-w,* as highlighted by the cluster analysis, are similar but notably distinct (particularly in clusters 2, 4, 6, and 7). Thus, these results could suggest that Gpr1 impinges on both PKA-dependent and PKA-indepen-dent signaling pathways. Alternatively, the steady-state differences between *gpr1* and *GPR1* strains at the onset of the experiment could render the strains differentially responsive to glucose. This issue could be resolved by appropriate conditional alleles in *GPR1* and *TPK*.

### Ras, Gpa2, and Glucose Induce Genes in Mass Accumulation and Repress Genes in Respiration and Mitochondrial Function

We have addressed the nature of the genes regulated by glucose and Ras2 in two different but related ways. First, we asked how those genes that have been annotated as performing related functions behave on average over the set of experiments. Second, we have determined whether genes performing a common function are significantly overrepresented in any cluster of coexpressed genes. Both approaches give essentially the same results.

**Figure 5:**
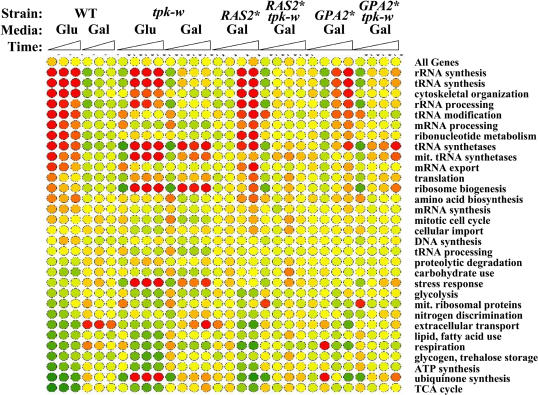
Functional Analysis of Glucose- and Ras-Induced Expression Changes The average expression levels of genes grouped by the functional category listed on the right in the indicated strains over the 1-h time course are indicated by color (red, induced; green, repressed; yellow, unchanged). Values are relative to the expression level in strain Y2864 prior to glucose addition. The Functional Classification Catalog was obtained from MIPS at http://mips.gsf.de/proj/yeast/CYGD/db/index.html. Functional group analysis was performed using the ratio of vector magnitudes (Kuruvilla et al. 2002). The computer source code was derived from http://www-schreiber.chem.harvard.edu. Strains: Y2864 (WT), Y2872 *(tpk-w),* Y2866 (RAS2*), Y2873 (RAS2* tpk-w), Y2876 (GPA2*), Y2897 (GPA2* tpk-w).

In Figure 5, we present the average level of expression of all the genes associated with the indicated function (as annotated by the Munich Information Center for Protein Sequences [MIPS] program) relative to that at time 0 in the wild-type strain. As evident, genes required for translation are upregulated by glucose and activation of Ras2 or Gpa2. This includes genes for RNA polymerase I and III subunits, cytoplasmic tRNA synthetases, rRNA and tRNA processing enzymes, translation initiation factors, and, to a slightly lesser degree, ribosomal proteins. Similarly, genes for these functional categories are highly enriched in those clusters in which expression increases following addition of glucose to wild-type or *tpk-w* cells or following activation of Ras2 or Gpa2 (see Table 1). Thus, a major portion of the transcriptional restructuring following glucose addition is directed toward enhancement of the translational machinery. Somewhat surprisingly, though, this is induced not solely by increased metabolism, but at least in part by a direct response to a signaling circuit, which is mediated at least in part by Ras2.

On the other side of the coin, genes involved in oxidative respiration, including components of the TCA cycle, oxidative phosphorylation apparatus, and ubiquinone (CoQ) synthesis, and all the genes required solely for gluconeogenesis are significantly downregulated both by glucose addition and by activation of Ras or Gpa2. These functional categories of genes are significantly overrepresented in that class of coexpressed genes that are downregulated in all conditions tested (class V). Thus, Ras2-dependent and Ras2-independent repression pathways redundantly regulate the restructuring associated with conversion from respiration to fermentation.

Several groups of genes appear to be regulated by glucose exclusively through a PKA-dependent pathway. These are genes repressed by Ras2 or Gpa2 and by glucose in the wild-type strain, but not in the *tpk-w* strain (class VI), and include those involved in carbohydrate storage (trehalose and glycogen) and, to a large extent, in ubiquinone synthesis. A number of genes exhibit induction by glucose in an exclusively Ras2-dependent fashion and include genes involved in ribosome biogenesis.

Reciprocally, a number of genes exhibit induction by glucose in a completely Ras-independent fashion. As noted in Figure 2, expression of members of class III increases monotonically following glucose addition, in contrast to the pattern seen with genes in other induction classes, in which an initial rapid increase in expression following glucose addition is followed by an immediate stabilization or downshift. This may indicate that these genes are upregulated as a consequence of the metabolic changes or growth acceleration attendant on glucose addition. The enrichment of genes involved in DNA replication in this category is consistent with this hypothesis.

### Identification of Potential Transcription Factors Mediating the Response to Ras2 Activation

We have used a number of computational approaches to identify potential regulatory sequences and regulatory factors responsible for changes in gene expression in response to glucose and/or Ras2 activation. All of these approaches are based on the assumption that genes exhibiting a common expression pattern over all the experiments are more likely to share a common regulatory sequence or respond to a common transcription factor (see Supporting Information).

Several motifs (RRPE, PAC) and transcription factor-binding sites (Sfp1, Rap1, Fhl1) are associated with the class of genes induced by glucose through both a Ras-dependent and a Ras-independent pathway. Rap1- and Fhl1-binding sites have previously been associated with ribosomal protein genes (Lieb et al. 2001; Lee et al. 2002), and the enrichment of these sites in this class represents the high proportion of ribosomal protein genes in the clusters comprising this class. Similarly, the RRPE and PAC motifs have been associated with genes encoding elements of the translational machinery and with genes that are upregulated following overexpression of Sfp1 (Hughes et al. 2000; Wade et al. 2001; Jorgensen et al. 2002). Thus, these three transcription factors and their associated motifs are potential loci through which glucose and/or Ras activates transcription of translation-related genes.

To evaluate whether the predicted motifs mediate Ras-activated transcription, we inserted each motif upstream of a reporter gene lacking any other upstream activation sequence (UAS) and then introduced the individual constructs into strains containing the inducible RAS2* or GPA2* alleles. As a positive control, we examined expression of the *RPS18B* promoter/enhancer region when it was fused to the reporter construct. As evident in Table 2, activation of Ras2 or Gpa2 resulted in a 3-fold increase in expression of the reporter construct, consistent with the observation that expression of this gene increased following induction of either RAS2* or GPA2* in our genome-wide expression analysis. Having confirmed the ability of this system to detect Ras-responsive promoters, we examined the ability of the Rap1-binding site or the RRPE or PAC element to enhance transcription in response to activation of the Ras pathway. As noted in Tables 2, 3, and 4, both the Rap1-binding site and the RRPE element yielded strong enhancer activity, especially when present in multiple copies. In contrast, the PAC element exhibited no enhancer activity. Further, the Rap1 enhancer activity increased modestly but consistently in glucose versus glycerol medium and following activation of Ras2 or Gpa2. Activation of Ras2 or Gpa2 also consistently yielded increased expression driven by the RRPE element. Finally, an MCB element provided modest enhancer activity that was further stimulated by growth on glucose but not by activation of Ras2 or Gpa2. This is consistent with the expression pattern of genes in the cluster in which the MCB motif is enriched.

**Table 2:**
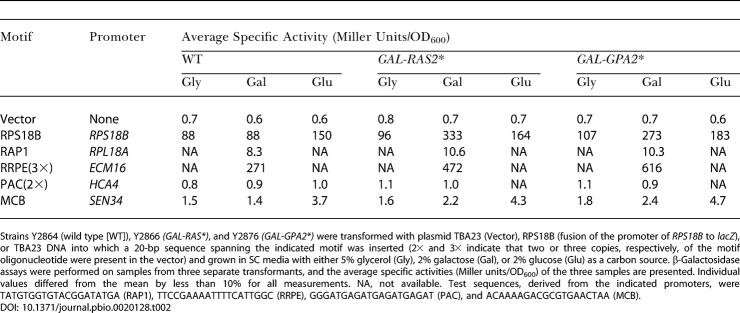
Functional Analysis of Motifs: Potential Activator Elements

Strains Y2864 (wild type [WT]), Y2866 (GAL-RAS*), and Y2876 (GAL-GPA2*) were transformed with plasmid TBA23 (Vector), RPS18B (fusion of the promoter of *RPS18B* to *lacZ*), or TBA23 DNA into which a 20-bp sequence spanning the indicated motif was inserted (2× and 3× indicate that two or three copies, respectively, of the motif oligonucleotide were present in the vector) and grown in SC media with either 5% glycerol (Gly), 2% galactose (Gal), or 2% glucose (Glu) as a carbon source. β-Galactosidase assays were performed on samples from three separate transformants, and the average specific activities (Miller units/OD_600_) of the three samples are presented. Individual values differed from the mean by less than 10% for all measurements. NA, not available. Test sequences, derived from the indicated promoters, were TATGTGGTGTACGGATATGA (RAP1), TTCCGAAAATTTTCATTGGC (RRPE), GGGATGAGATGAGATGAGAT (PAC), and ACAAAAGACGCGTGAACTAA (MCB)

**Table 3:**
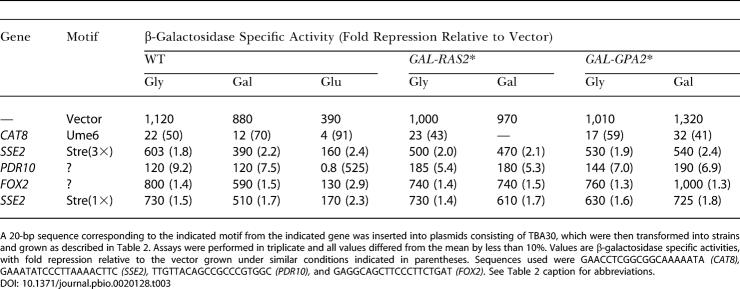
Functional Analysis of Motifs: Potential Repressor Elements

A 20-bp sequence corresponding to the indicated motif from the indicated gene was inserted into plasmids consisting of TBA30, which were then transformed into strains and grown as described in Table 2. Assays were performed in triplicate and all values differed from the mean by less than 10%. Values are β-galactosidase specific activities, with fold repression relative to the vector grown under similar conditions indicated in parentheses. Sequences used were GAACCTCGGCGGCAAAAATA *(CAT8),* GAAATATCCCTTAAAACTTC *(SSE2),* TTGTTACAGCCGCCCGTGGC *(PDR10),* and GAGGCAGCTTCCCTTCTGAT *(FOX2)*. See Table 2 caption for abbreviations

**Table 4:**
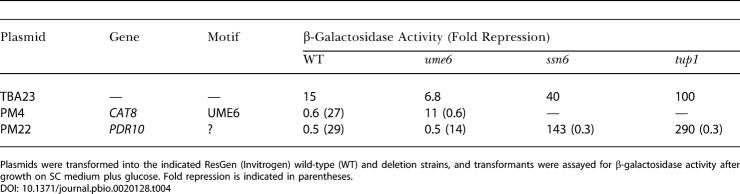
Functional Analysis of Motifs: *PDR10* Element Is Not Ume6 Dependent

Plasmids were transformed into the indicated ResGen (Invitrogen) wild-type (WT) and deletion strains, and transformants were assayed for β-galactosidase activity after growth on SC medium plus glucose. Fold repression is indicated in parentheses

Several motifs were identified as correlated with repression by glucose and by Ras2 or Gpa2. These included binding sites for Rpn4, Ume6, Hap2/3/4, and Msn2/4 as well as several sequences of unknown association. We tested several of these motifs for their ability to mediate glucose- or Ras-induced transcriptional repression by inserting them between the *CYC1* UAS and the promoter of a *CYC1*-*lacZ* reporter construct and examining expression under different growth conditions. Most of the known elements manifested modest repression activity that was not enhanced by growth on glucose or by Ras or Gpa2 activation. However, multiple copies of an Ume6-like element from *PDR10* elicited strong glucose-enhanced repression activity. As evident from Table 3, the element caused 5- to 10-fold repression when cells were grown in glycerol and 500-fold repression when cells were grown in glucose. While this element exhibits some similarity to a Ume6-binding site, it does not mediate repression by Ume6. As noted in Table 4, deletion of *UME6* (or *RPN4, MIG1, MIG2, MSN4, PHD1, RGM1, STD1, RIM101, SFL1,* or *NRG1;* data not shown) did not alleviate the repressive effects of this element, although this deletion eliminated repression effected by a known Ume6-binding site from *CAT8*. Repression by the *PDR10* site was alleviated by deletion of *TUP1* or *SSN6*. Thus, this element likely functions by recruiting the Tup1/Ssn6 repressor complex to the promoter through a specific DNA binding factor intermediate. Given the lack of correspondence between the sequence of the element and known regulatory motifs, the element likely represents a novel glucose repression mechanism.

## Discussion

### Defining the Glucose Signal Transduction Pathway

Transcriptional regulation by glucose has been examined extensively by genetic and biochemical analyses of specific glucose-repressible and glucose-inducible genes as well as by global transcriptional analysis (DeRisi et al. 1997; Lutfiyya et al. 1998; Hughes et al. 2000; Wade et al. 2001; Jorgensen et al. 2002). These studies have highlighted pathways involved in connecting the presence of glucose with changes in the transcription state of the cell, particularly those pathways mediated by the Snf1/4 kinase and the Grr1 ubiquitin ligase (Carlson 1999; Johnston 1999). Similarly, previous studies have demonstrated that the Ras/PKA pathway responds to glucose addition and affects gene expression, implicating Ras/PKA as a mediator of the cell's response to glucose. However, the overall topology of the glucose signaling network in yeast and the extent to which these different branches contribute and interconnect have not been previously addressed. The approach described in this report, following an earlier conceptual framework (Roberts et al. 2000), provides a means of developing systematically a comprehensive topological map of the glucose signal network. Thus, this report is a first step in defining such a network.

**Figure 6:**
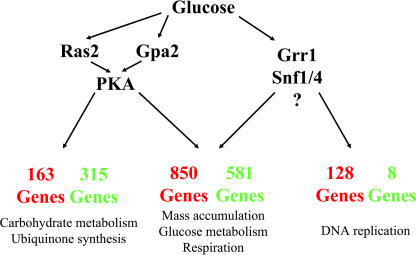
The Role of Ras and Gpa2 in Glucose Regulation of Transcription Diagram of information flow in glucose signal of transcription as deduced from global analysis of expression of genes in the strains used in this study. The number of genes regulated by each branch of the pathway, the nature of the regulation (red, induction; green, repression), and some of the functional categories of genes enriched in each branch are indicated.

In this study, we have shown that most of the changes in transcription attendant on glucose addition can be recapitulated by activation of Ras2 or Gpa2. Thus, most of the glucose-induced changes in gene expression can be mediated by Ras2 and Gpa2. This is surprising since most transcriptional responses to glucose, particularly glucose repression, have been associated with Ras-independent mechanisms (Gancedo 1998). In fact, though, since most of the glucose-induced transcriptional changes are also observed in a strain lacking a cAMP-responsive PKA, most of the glucose effects can also be mediated by a Ras/PKA-independent pathway. Thus, a minimal topology for the signaling pathway for modifying transcription in response to glucose comprises (1) redundant signaling pathways for repression and induction of the majority of genes, (2) a Ras/PKA-independent branch, and (3) a branch that is solely mediated by Ras/PKA (Figure 6). Whether the redundant pathways reconverge at specific transcription factors or at the promoters of genes themselves remains to be determined. In addition, the relative contributions of known glucose regulatory circuits to the Ras-independent pathways, such as those mediated by Snf1/4 and Grr1, have not been determined. Studies similar to those described here for Ras are currently in progress with other contributing pathways.

A redundant pathway for glucose signaling is consistent with previous observations suggesting that while activation of Ras/PKA elicits substantial changes in growth and carbohydrate metabolism in the cell, most of those changes can be effected even in the absence of an active Ras/PKA pathway. Cameron et al. (1988) constructed and analyzed *tpk-w* strains of yeast, which, as noted above, contain a PKA that is unresponsive to changes in cAMP levels. The authors found that *tpk-w* strains not only reverse all the phenotypes of *bcy1* strains, but also regain the ability to respond to glucose depletion and readdition (glycogen accumulation, sporulation, etc.) in a timely and appropriate manner. Thus, the authors concluded that, while Ras/PKA could affect the cell's growth response to nutrients, one or more cAMP-indepen-dent pathways regulate the cell's response to nutrient availability. Under circumstances in which the cAMP signaling pathway is maintained at a moderate but constant level, this additional pathway(s) is sufficient for normal nutrient regulation. The presence of redundant glucose signaling in yeast could explain these earlier results.

Most of the changes in transcription measured in these experiments likely result from the activity of a signal transduction pathway responsive to glucose, rather than from indirect effects due to changes in growth rate or metabolism. We saw the same global response whether the induction protocol was galactose addition in a *gal1* background or addition of the gratuitous inducer β-estridiol to a strain with Ras2 or Gpa2 activation driven by a lexA-ER-VP16 chimeric transcription factor (Louvion et al. 1993). Thus, the method of induction does not influence the results, ruling out any metabolic influences on the response. In addition, the glucose-induced transcription effects are observed early, likely prior to substantial reprogramming of the metabolic machinery of the cell. Transcriptional responses to glucose addition at later timepoints are often in opposite polarity to those at early timepoints, which suggests that the cell adapts its transcriptional response to the new conditions and emphasizes the importance of kinetic analysis in order to capture the structure of the signaling network under initial conditions.

Several patterns of expression are not explained in a straightforward manner by the network depicted in Figure 6. For instance, genes in class IV are induced by activation of either Ras2 or Gpa2 and by glucose addition to wild-type cells, but are repressed by glucose addition to *tpk-w* cells. Genes of class VII show the inverse behavior. One possible explanation is that the Ras-dependent and Ras-independent pathways have opposite effects on expression of these sets of genes. Alternatively, the physiology of the *tpk-w* cells may be significantly different than that of wild-type cells under initial conditions, such that the baseline expression of some genes at time 0 is significantly different in the two strains. In fact, the transcriptional profile of the *tpk-w* strain at time 0 is significantly different from that of the isogenic wild-type strain. This latter explanation may account for the behavior of genes in class VIII, which exhibit repression only by glucose addition to *tpk-w* cells. The behavior of class VIII genes may also suggest that some transcription factor activity or promoter activity is saturable, an hypothesis explored in more depth elsewhere (Lin et al. 2003).

### Ras and Gpa Signal Exclusively through PKA

We used epistasis analysis to define the functional topology of the Ras2 and Gpa2 branch of the glucose signaling pathway. That is, we examined the transcriptional consequences of activating Ras2 or Gpa2 in a background lacking a cAMP-responsive PKA. Since the readout of this experiment is the entire transcriptome of the cell, we can determine whether any gene is regulated by Ras in a PKA-independent fashion without knowing a priori what that gene might be. Our results demonstrate that all transcriptional effects of Ras2 and of Gpa2 are mediated by PKA. Previous studies have suggested that in certain strains Ras2 can activate the filamentous growth MAP kinase pathway (Mosch et al. 1996, 1999). Our results clearly indicate that in the strain examined grown under the conditions described, no such connection between Ras and the MAP kinase pathway exists. Further, identical epistasis experiments performed with diploid Σ1278 strains yielded the same result (data not shown). Thus, while Ras exerts PKA-independent effects on the yeast cell, all the transcriptional effects of Ras proceed through PKA.

Substantial information has accumulated to suggest that, like Ras2, activated Gpa2 stimulates adenylyl cyclase, leading to an increase in cellular cAMP levels (Kubler et al. 1997; Lorenz and Heitman 1997), although recent evidence suggests that Gpa2 might activate PKA directly (J. P. Hirsch, personal communication). Genetic epistasis data to date indicate that to activate PKA, Ras2 and Gpa2 proteins act in redundant parallel pathways, rather than in sequential steps in the same pathway (Xue et al. 1998). However, whether activation of PKA is the sole activity of Gpa2 is not known. Our results indicate that, like Ras2, all of the transcriptional effects of Gpa2 are mediated by PKA. Consistent with that conclusion, we do not detect any group of genes whose expression is altered by activation of Gpa2 and is not also similarly altered by activation of Ras2. We do note that the intensity of transcriptional response following activation of Gpa2 is approximately half that seen following activation of Ras, suggesting that while both proteins function in similar roles, they have quantitatively different effects.

### Potential Transcriptional Network

Various computational approaches identified a number of sequence motifs and transcription factors through which glucose and Ras2 or Gpa2 might be modulating transcription. The presence of the previously identified RRPE and PAC motifs is strongly correlated with genes induced following Ras2 activation. The pattern of genes induced by Ras2 closely resembles that of genes induced by increased expression of the Sfp1 transcription factor (Jorgensen et al. 2002). We find that RRPE acts as a strong enhancer element in reporter gene constructs and that its enhancer activity is increased following activation of Ras2. Sfp1 contains several PKA consensus phosphorylation sites. However, evidence that Sfp1 acts directly through PAC/RRPE or that Sfp1 is the locus of PKA-induced activation is not yet available. Our studies also returned a strong correlation between genes induced by Ras2 and those containing Rap1-binding sites in their promoters, confirming the previously identified role of Rap1 in mediating PKA regulation of ribosomal protein gene expression (Klein and Struhl 1994; Neuman-Silberberg et al. 1995). Recent results suggest that Rap1 binding to promoter sites serves to recruit the histone acetyl transferase Esa1 and that Rap1 binding is constitutive, but Esa1 recruitment is modulated by growth conditions (Reid et al. 2000; Rohde and Cardenas 2003). Thus, PKA may affect the interaction of Rap1 and Esa1, an hypothesis currently under investigation.

Our computational studies confirmed the presence of a number of motifs associated with glucose regulation and PKA, including the STRE element as well as binding sites for Hap2/3/4, Ume6, and Rpn4. Recent data have shown that PKA directly affects the nuclear localization of Msn2, one of the transcription factors that acts through STRE, but that PKA does so through a mechanism independent of the one responsive to environmental stress (Gorner et al. 2002). Thus, the convergence of the glucose signal and the stress response signal on this transcription factor could account in part for the overlap of transcriptional response of the cell to glucose depletion and other forms of environmental stress (Gasch et al. 2000; Causton et al. 2001). We also identified a motif associated with genes repressed by glucose through Ras-dependent and Ras-independent pathways. This motif provokes repression in reporter constructs that is substantially enhanced in growth on high levels of glucose, although the repression does not appear to be altered by PKA activation. While the motif bears resemblance to both Ume6 and Rpn4, it does not mediate repression by either factor, since deletion of either gene does not alleviate glucose-dependent repression by the motif. Thus, we have identified a novel glucose regulatory motif through these computational approaches. Further analysis of the many other motifs identified in this study could yield additional novel regulatory elements.

## Materials and Methods

### Strains

All strains used in this study were derived from W303–1B and are listed in Table 5. *tpk-w* alleles were isolated as described by Cameron et al. (1988) and confirmed by sequencing and retransformation of the mutant *tpk2* allele. Construction of the galactose-inducible *RAS2* ^G19V^ allele has been described by Fedor-Chaiken et al. (1990). The activated allele of *GPA2* (*GPA2* ^Q300L^) was placed under the control of the *GAL10* promoter (plasmid B2364), digested with ClaI, and integrated into the *LEU2* locus of Y2864 and Y2895 to obtain strains Y2876 and Y2897. Yeast Consortium Deletion Strains created in the BY4742 background (*MAT*α *his3*Δ *leu2*Δ *lys2*Δ *ura3*Δ) were obtained from Research Genetics (Invitrogen, Carlsbad, California, United States). The *ho*Δ strain was used as a wild-type control**.**

**Table 5:**
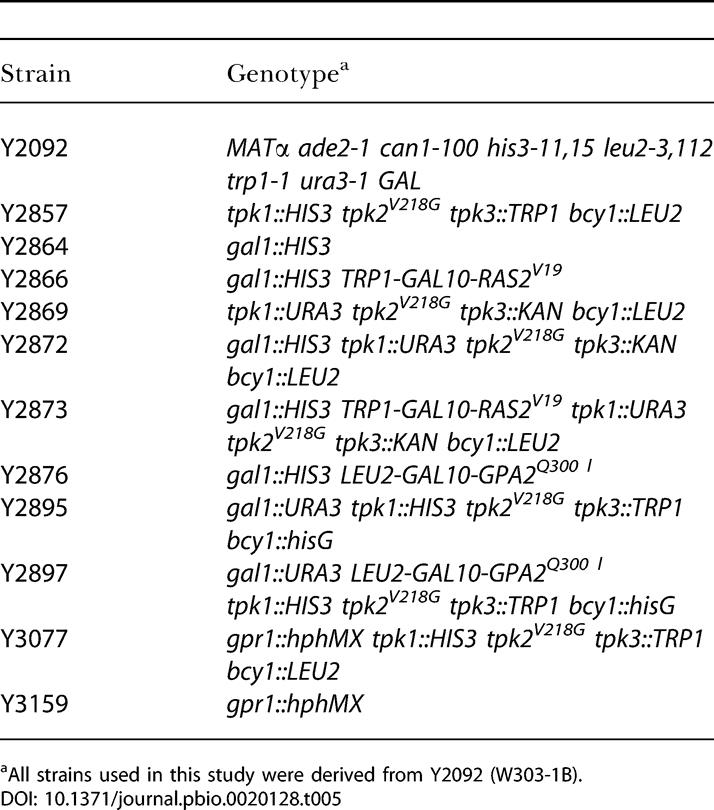
Strains Used in This Study

^a^All strains used in this study were derived from Y2092 (W303-1B)

### Cell Growth

Cells were streaked on YEPD plates and grown for 2–3 d at 30°C. Fresh colonies were inoculated into synthetic complete (SC) medium supplemented with 3% glycerol as the only carbon source. Cells were grown at 30°C and shaken at 200 rpm to an OD_600_ of 0.25 (budding index, approximately 20%), at which time an aliquot of cells was removed as the time-0 control. Glucose or galactose was then added to 2% in the remaining culture and aliquots (40 ml) of cells were collected at 20, 40, and 60 min following sugar addition. Cells were mixed with 100 ml of prechilled water and quickly spun down by centrifugation at 2,500 rpm for 3 min at 4°C.

### RNA Isolation, Labeling, and Hybridization

Cell pellets were lysed in TRI reagent (Molecular Research Center, Cincinnati, Ohio, United States) by vortexing with glass beads for 3 min. After a 5-min incubation at room temperature, 0.2 ml of chloroform per 1 ml of TRI reagent was added and mixed well with the homogenate. After centrifugation at 14,000 rpm for 15 min at 4°C, the upper aqueous phase was removed and precipitated with equal volume of isopropanol. RNA pellets were washed with 75% ethanol, air-dried, and dissolved in water. mRNA was purified from the total RNA with oligotex (Qiagen, Valencia, California, United States).

First-strand cDNA was synthesized from mRNA using HPLC-purified T7-(dT)_24_ primer (Genset, San Diego, California, United States) and SuperScript II RT (Invitrogen). Second-strand cDNA was synthesized using DNA ligase (10 U), DNA polymerase I (40 U), and RNase H (2U) from Invitrogen. Biotin-labeled cRNA was made with a BioArray HighYield RNA transcript labeling kit (Enzo Diagnostics, Farmingdale, New York, United States) and purified using an RNeasy mini-kit (Qiagen). The cRNA was fragmented, mixed with control cRNA cocktail, and hybridized to yeast genome S98 array (Affymetrix, Santa Clara, California, United States) for 16 h in a 45°C oven rotating at 60 rpm. The probe arrays were washed and stained using the GeneChip Fluidics station 400 (Affymetrix) and scanned at 570 nm with the Agilent GeneArray scanner (Affymetrix).

For each experiment, we examined multiple timepoints, and for samples of significant interest we performed the experiment in triplicate. For initial analysis, we used MicroArray Suite 5.0 software (Affymetrix) to determine whether the hybridization signal for a gene was reliable and incorporated in our analysis only those measurements that were judged present, which generally included greater than 90% of the gene measurements in any one sample, with greater than 75% of all genes yielding reliable values over all the experiments. We also eliminated from our initial analysis those genes that were induced more than 3-fold in the *gal1* strains by addition of galactose (25–30 genes, depending on the experiment). All experiments were normalized to the same total signal intensity. Data for all experiments can be obtained from Tables S1, S2, and S3 or at http://www.molbio.princeton.edu/labs/broach/microarray.htm.

### Computational Methods

#### Expression clustering and motif discovery

Partitional clustering of gene expression data was performed using the Qtclust algorithm (Heyer et al. 1999), which creates a partitioning of genes into nonoverlapping clusters. Not all genes are assigned to clusters, as the members of each cluster are guaranteed to have a minimal intergene Pearson correlation (in our case, 0.75). In order to identify putative transcription factor-binding sites, the members of each cluster were used to search for common DNA sequence motifs in their 5′ upstream region using the AlignACE algorithm (Tavazoie et al. 1999). For each cluster, three independent motif searches were performed. The resulting pool of approximately 5,000 motifs contained significant redundancy, as many known binding sites were identified multiple times. Using a motif similarity measure in the CompareACE algorithm (Hughes et al. 2000), we clustered all the motifs into a largely nonredundant set of 251 members. In order to obtain a more “coarse-grained” view of genome-wide expression patterns, the original 144 clusters were combined by hierarchical clustering (Eisen et al. 1998) of their mean expression profiles, yielding the eight classes discussed in the paper.

#### Known transcription factor binding sites

We assembled a set of weight matrices corresponding to 45 well-characteri*z*ed Saccharomyces cerevisiae transcription factors. These matrices were constructed from a mix of experimentally determined binding sites, augmented with extensive expression and chromatin IP-derived data (Lee et al. 2002). To this list, we added three weight matrices (PAC, RRPE, A/T_repeat), which had strong computational evidence for being real transcription factor-binding sites.

#### Statistical analysis

To determine the statistical significance of functional enrichments in expression clusters, we used the hypergeometric distribution to quantify the chance probability of obtaining the observed overlap between an expression cluster and any of the 200 functional categories defined in the MIPS database (Tavazoie et al. 1999; Mewes et al. 2002). The hypergeometric distribution was also used to quantify the probability of obtaining the observed overlap between expression clusters and the set of 300 genes with the highest-scoring occurrences of a motif in their 5′ upstream region.

### Reporter Gene Analysis

Oligonucleotides containing motif sequences from selected promoters were cloned into the XhoI site of the *CYC1*-*lacZ* reporter vectors pTBA23 and pTBA30, as described previously (Mead et al. 1996). The former vector contains the *CYC1* promoter and UAS, with the XhoI site residing between them, and the latter vector contains only the *CYC1* promoter. Assays were performed on three separate transformants for each construct, grown as indicated. Results of β-galactosidase assays differed by less than 10% for triplicate measurements (Gailus-Durner et al. 1997).

## Supporting Information

Table S1 Gene Expression Patterns Following Glucose Addition and Ras2 and Gpa2 Activation Strains were as follows: Wild-type = Y2864 (*MATα ade2-1 can1-100 his3-11,15 leu2-3,112 trp1-1 ura3-1 gal1::HIS3*); *tpk-w* = Y2872 (*MATα ade2-1 can1-100 his3-11,15 leu2-3,112 trp1-1 ura3-1 gal1::HIS3 tpk1::URA3 tpk2^V218G^ tpk3::KAN bcy1::LEU2*); RAS* = Y2866 (*MATα ade2-1 can1-100 his3-11,15 leu2-3,112 trp1-1 ura3-1 gal1::HIS3 TRP1-GAL10-RAS2^V19^*); *RAS tpk-w* = Y2873 (*MATα ade2-1 can1-100 his3-11,15 leu2-3,112 trp1-1 ura3-1 gal1::HIS3 TRP1-GAL10-RAS2^V19^ tpk1::URA3 tpk2^V218G^ tpk3::KAN bcy1::LEU2*); GPA2* = Y2876 (*MATα ade2-1 can1-100 his3-11,15 leu2-3,112 trp1-1 ura3-1 gal1::HIS3 LEU2-GAL10-GPA2^Q300L^*) *GPA tpk-w* = Y2897 (*MATα ade2-1 can1-100 his3-11,15 leu2-3,112 trp1-1 ura3-1 gal1::HIS3 LEU2-GAL10-GPA2^Q300L^ tpk1::HIS3 tpk2^V218G^ tpk3::TRP1 bcy1::hisG*) .
Experimental conditions were as follows. Cells were grown in SC medium plus 3% glycerol to A_600_ = 0.3. Glucose or galactose was added to 2%, and samples were removed at 0, 20, 40 and 60 min following the addition.
Microarrays were performed as follows. RNA was isolated and labeled as described in Materials and Methods and hybridized to Affymetrix yeast genome S98 arrays.
Data presentation is as follows. The first five columns provide the gene name (if known), the *Saccharomyces* Genome Database (SGD) gene designation, the MIPS functional category, and the function of the gene product, and the Affymetrix probe was set for that gene. For each time sample, the first column provides the normalized intensity values and the second column provides the determination from the MicroArray Suite 5.0 software as to whether the value was significant (P), insignificant (A), or indeterminate (M). The table is in a tab-delimited text format.
(1.99 MB TXT).

Table S2 Gene Expression Patterns Following Glucose Addition to *gpr1* and *gpr1 tpkw* Strains Strains were as follows: Y2092 = *MATα ade2-1 can1-100 his3-11,15 leu2-3,112 trp1-1 ura3-1*; Y3159 = *MATα ade2-1 can1-100 his3-11,15 leu2-3,112 trp1-1 ura3-1 gpr1::hphMX*; Y2857 = *MATα ade2-1 can1-100 his3-11,15 leu2-3,112 trp1-1 ura3-1tpk1::HIS3 tpk2^V218G^ tpk3::TRP1 bcy1::LEU2*; Y3077 = *MATα ade2-1 can1-100 his3-11,15 leu2-3,112 trp1-1 ura3-1tpk1::HIS3 tpk2^V218G^ tpk3::TRP1 bcy1::LEU2 gpr1::hphMX*.
Experimental conditions were as follows. Cells were grown in SC medium plus 3% glycerol to A_600_ = 0.3. Glucose was added to 2%, and samples were removed at 0, 20, 40 and 60 min following the addition.
Microarrays were performed as follows. Reference samples (RNA from 0 timepoint in each experiment) were labeled with Cy3, and each test sample (RNA from subsequent timepoints) was labeled with Cy5, mixed with the corresponding reference sample, and hybridized to cDNA microarrays printed in-house.
Data presentation is as follows. Values are ratios of RNA levels for each gene at the indicated timepoint relative to the level for that gene at time 0 in that particular experiment.
(477 KB TXT).

Table S3 Members of Gene Expression Classes The set of genes, specified by their SGD designation, comprising each of the gene expression classes listed in Table 1 and diagrammed in Figure 2, is listed for each class. The table is a tab-delimited text file.
(29 KB TXT).

## Abbreviations

cAMP: cyclic AMP
GO: Gene Ontology
MIPS: Munich Information Center for Protein Sequences
PKA: cAMP-dependent protein kinase A
SC: synthetic complete
SGD: *Saccharomyces* Genome Database
UAS: upstream activation sequence
